# Effects of Probiotics on the Growth Performance, Antioxidant Functions, Immune Responses, and Caecal Microbiota of Broilers Challenged by Lipopolysaccharide

**DOI:** 10.3389/fvets.2022.846649

**Published:** 2022-02-21

**Authors:** Yang Yu, Qing Li, Xinfu Zeng, Yinglei Xu, Kan Jin, Jinsong Liu, Guangtian Cao

**Affiliations:** ^1^Key Laboratory of Applied Technology on Green-Eco-Health Animal Husbandry of Zhejiang Province, Zhejiang Province Engineering Laboratory for Animal Health and Internet Technology, College of Ani-mal Science and Technology, College of Veterinary Medicine, Zhejiang A & F University, Hangzhou, China; ^2^Zhejiang Vegamax Biotechnology Co., Ltd., Anji, China; ^3^College of Standardisation, China Jiliang University, Hangzhou, China

**Keywords:** probiotics, lipopolysaccharide, immunity response, caecal microbiota, broiler

## Abstract

We aimed to study the effects of dietary *Bacillus coagulans* (*B. coagulans*) and *Lactobacillus* p*lantarum* (*L. plantarum*) on broilers challenged by *Escherichia coli* lipopolysaccharide (LPS). One-day-old Cobb 500 chicks (360) were divided randomly into three treatment groups for 47 days: no supplementation (control, CON), *B. coagulans* supplementation (BC), and *L. plantarum* supplementation (LA). Broilers were routinely fed for 42 days and intraperitoneally injected with 500 μg LPS per kg body weight at 43, 45, and 47 days of age, respectively. Samples were collected 3 h after the last injection. At 1–21 days of age, the ADG in the BC and LA groups was higher than that in the CON group, and the feed to gain ratio (F/G) in the BC group was significantly decreased (*P* < 0.05). Compared with that in CON birds, the ADG was increased and the F/G was decreased in the BC and LA birds at 22–42 and 1–42 days of age, respectively (*P* < 0.05). After LPS stimulation, the endotoxin (ET), diamine oxidase (DAO), and D-lactic acid (D-LA) levels in the BC group were lower than those in the CON group (*P* < 0.05). The IgY, IgA, and IgM contents in the BC group and the IgY and IgM contents in the LA group were higher than those in the CON group (*P* < 0.05). The pro-inflammatory factor and interferon-β (IFN-β) contents (*P* < 0.05) decreased, and the anti-inflammatory factor content in the serum (*P* < 0.05) increased in the BC and LA groups. Compared with the CON and LA treatments, the BC treatment increased the concentrations of glutathione peroxidase (GSH-Px), superoxide dismutase (SOD), and catalase (CAT), and decreased that of malondialdehyde (MDA) (*P* < 0.05). In contrast with the CON treatment, the BC and LA treatments increased the abundance of *Ruminococcaceae* and reduced that of *Desulfovibrio* (*P* < 0.05). Moreover, BC increased the abundance of beneficial bacteria. Overall, supplementation with *B. coagulans* and *L. plantarum* promoted the growth of broilers, improved their immunity and antioxidant capacity, and alleviated the LPS-stimulated inflammatory response by regulating the intestinal flora.

## Introduction

High human demand for poultry meat has led to intensive production, and intensive systems are particularly susceptible to production diseases such as oxidative stress, diarrhea, and enteritis (1, 2). In addition, inflammation associated with the innate immune responses is a common challenge for poultry farms and leads to significant economic losses (3). Such as GSH Px, SOD, and CAT are antioxidant enzymes and MDA is the product of lipid peroxidation. Recent a study found that excess cadmium (Cd) decreased SOD and CAT, increased MDA, and caused common carp liver oxidative stress (4). Another study demonstrated that the down-regulation of GSH-Px, SOD, and CAT, as well as the up-regulation of MDA took part in Cd-induced oxidative stress; meanwhile the up-regulation of TNF-α and the down-regulation of IL-10 took part in Cd-induced immunosuppression in common carp gills (5). Ammonia gas exposure increased IL-1β and IL-6, and caused immunotoxicity in broiler spleens (6). Probiotics and prebiotics initiate resistance to bacterial colonization and enhance the mucosal immunity of the host, thereby minimizing the burden of pathogens (7, 8). Previous studies have demonstrated the safety of *Bacillus coagulans* and its potential for food and medical applications (9, 10). Moreover, Benbara et al. (11) have demonstrated the safety of *Lactobacillus plantarum* (*Lactobacillus plantarum S27*) and its beneficial effects on the performance of chickens.

Lipopolysaccharide (LPS), the main component of the outer membrane of Gram-negative bacteria, leads to the production of inflammatory mediators through toll-like receptors and is an effective activator of the innate immune response. Therefore, it is widely used in the establishment of animal immune stress models (12, 13). Several studies have indicated that probiotics affected the growth performance and immune status of LPS-challenged animals. For example, *yeast* and its derivatives had improved LPS-induced changes in white the counts of blood cells, lymphocytes, and monocytes levels in broilers (14). Probiotics also reduced LPS-induced changes in the body weight of female mice 48 h post-treatment. Moreover, probiotic treatment prevented LPS-induced increases in pro- and anti-inflammatory (IL-1β, TNF-α, IL-6) peripheral cytokines at 8 h following LPS treatment, reduced the mRNA expression of central cytokine in the hypothalamus, hippocampus, and prefrontal cortex (PFC), and prevented LPS-induced changes in the gut microbiota (15). Deng et al. (16) reported that the administration of probiotic strains *Bacillus licheniformis* or *Bacillus subtilis* improved intestinal function, ameliorated the inflammation response, and modulated the microflora after LPS-induced acute inflammation in rats. However, the effects of probiotics on the intestinal microflora of broilers have been less studied in LPS-induced models. Thus, the aim of this study was to elucidate the beneficial effects of probiotics (*B. coagulans* and *L. plantarum*) on LPS-induced broilers by the determination of relevant indices.

## Materials and Methods

### Birds' Management

**Figure 1:**
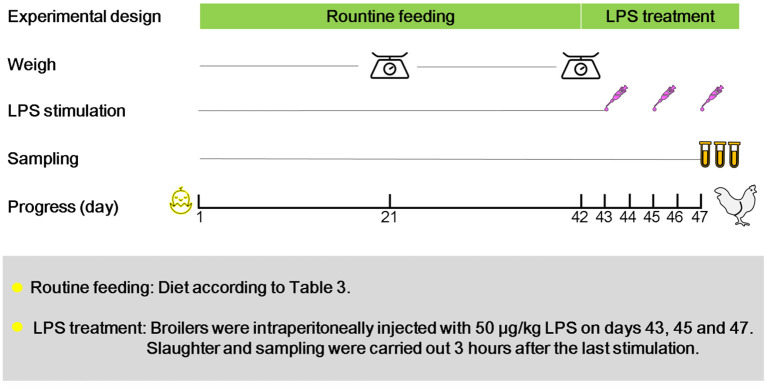
Experimental design. The experiment was divided into 3 groups: birds fed basal diet (CON); birds fed basal diet supplemented with 5 × 10^9^ cfu/kg *Bacillus coagulans* (BC); birds fed basal diet supplemented with 5 × 10^8^ cfu/kg *Lactobacillus plantarum* (LA). All broilers were raised routinely in 1–42 days old, the death and feed intake were recorded daily. Weighing and statistics were conducted on the 21st and 42nd day.

A total of 360 one-day-old Cobb broilers (half male and half female) were purchased from a local commercial company and randomly divided into the following three groups: (1) birds fed basal diet (CON); (2) birds fed basal diet supplemented with 5 × 10^9^ cfu/kg *Bacillus coagulans* (BC); (3) birds fed basal diet supplemented with 5 × 10^8^ cfu/kg *Lactobacillus. plantarum* (LA). Each group consisted of 6 replicates with 20 broilers per replicate. The experimental period was 47 days, and during this period, birds could feed and drink freely. The initial brooding temperature was 35°C, which was gradually reduced to 26 ± 1°C by 2°C per week until the end of the trial. The death and feed consumption were recorded daily for 42 days. On days 43, 45, and 47, all broilers were intraperitoneally injected with 50 μg/kg of LPS (Figure 1). Samples were collected 3 h after the last stimulation. The basic diet composition and nutrition level followed NRC 1994 (Table 1). The strains (*Bacillus coagulans* and *Lactobacillus. Plantarum*) and lipopolysaccharide used in this trial are commercially available.

**Table 1:** Raw material composition and nutritional level of basic dietary (air-dry basis).

**Items**	**1–21d**	**22–42d**
**Ingredients (%)**		
Corn	61.80	65.60
Soybean meal	22.50	17.55
Extruded soybean	8.45	10.00
Import fish meal	3.00	3.00
CaHPO4	1.66	1.45
Limestone	1.10	1.00
NaCl	0.32	0.30
DL- methionine	0.16	0.10
L- lysine	0.01	
Premix^*^	1.00	1.00
Total	100.00	100.00
**Nutrition levels**		
Metabolizable energy (MJ/kg)	12.45	12.70
Crude protein	21.00	19.20
Lysine	1.15	0.95
Methionine	0.54	0.44
Calcium	0.99	0.89
Available phosphorus	0.53	0.49

* *Premix is provided for feed per kg: VA 1,500 IU, VB_1_ 1.5 mg, VB_6_ 3.0 mg, VB_12_ 0.01 mg, VD_3_ 200 IU, VE 10 IU, VK 0.5 mg, Biotin 0.15 mg, D-pantothenic acid 10 mg, Folic acid 0.5 mg, Nicotinic acid 30 mg, Trace elements Cu, Fe, Zn, Mn, Se, I are 8, 80, 40, 60, 0.15, 0.18 mg respectively*.

### Sample Collection

At day 42, 6 birds per replicate were weighed. After 3 h of stimulation on day 47, euthanasia (Cervical dislocation was performed by an experienced operator) and sampling were conducted. Blood samples were collected from the vein under the wing and centrifuged at 3,000 × *g*, and the supernatant was separated and stored at −80°C. After euthanizing, Jejunal mucosa of broilers was removed and stored at −80°C for immune and antioxidant measuring. Further, the cecal contents were collected and immediately stored at −80°C for bacterial flora 16S ribosomal RNA (rRNA) sequencing. During the sampling process, the sampling tools and sample storage containers were sterile.

### Performance Evaluation

At the beginning of experiment, every bird was weighed and recorded for individual error reduction. At the end of experiment, the average daily feed intake (ADFI), average daily gain (ADG), and feed to gain ratio (F/G) during 1–21, 22–42, and 1–42 days of age were calculated according to the data recorded every day during the trial.

### Immunoglobulin Content

Referring to the specific manual, the contents of immunoglobulin A (IgA), immunoglobulin M (IgM), and immunoglobulin Y (IgY) in the serum and jejunal mucosa were tested with ELISA kits (Cusabio, Wuhan, China).

### Inflammatory Factor Level

Tumor necrosis factor-α (TNF-α), interferon-β (IFN-β), interleukin 1β (IL-1β), interleukin 6 (IL-6), and interleukin10 (IL-10) contents in the serum and jejunal mucosa were detected using specific ELISA kits (Nanjing Jiancheng, Nanjing, China) following the manufacturer's instructions.

### Mucosa Injury Indices

The glutathione peroxidase (GSH-Px), superoxide dismutase (SOD), malondialdehyde (MDA), antioxidant capacity (AOC), and catalase (CAT) contents in the serum were measured for antioxidation determination. The concentration of endotoxin (ET), diamine oxidase (DAO), and D-lactic acid (D-LA) in the serum was tested for determination intestinal injury. These targets were measured using commercial kits purchased from Nanjing Jiancheng (Nanjing, China) following the manufacturer's instructions.

### Cecal Microbial Sequencing

The process was entrusted to Shanghai Mega Biological Co., Ltd. The brief description of the process is as follows: the Illumina MiSeq platform (Illumina Inc., California, USA) was used for 300 paired-end sequencing in this study, wherein two samples of the same group were mixed into one biological sample. Then, the microbial genomic DNA of the cecum was extracted using a specific kit (Qiagen GmbH, Hilden, Germany). The primer used for the V3-V4 hypervariable region was 338F_806R (5′-ACTCCTACGGGAGGCACAG-3′; 5′-GGACTACHVGGGTWTCTAAT-3′). After PCR amplification and product purification, and PCR product quantification and homogenization, a PE library was constructed and Illumina (Illumina, San Diego, CA, USA) sequencing was performed. The Ultrafast sequence analysis (USEARCH) version 7.1 software was used for operational taxonomic unit (OTU) classification to classify the high-quality sequence valid tags obtained from the quality control according to the sequence similarity of 97%. Simpson and Shannon indices were used to analyze the alpha diversity in this study, and Student's *t*-test was used for to test the differences among the groups. Beta diversity analysis was conducted based on the OTU sequence similarity and community structure to compare the differences between different groups of samples, such as principal coordinates analysis (PCoA) and ternary phase diagrams. Unweighted_unifrac algorithm was adopted in PCoA. Microbial multivariate analysis was performed using the ANOVA algorithm to compare the significance of differences between groups.

### Statistical Analysis

One-way ANOVA and Duncan's test in IBM SPSS statistics (version 26.0, SPSS Inc., IIIinois, USA) were used for data analysis, and Graph Pad Prism 8.0 (Graph Pad Prism Inc., California, USA) was used for diagramming, wherein *P* < 0.05 meant significant difference and were marked with “^*^” in figures, and *P* < 0.01 meant significant difference and were marked with “^**^” in figures.

## Results

### Effects of Probiotics on the Growth Performance of Broilers Induced by LPS

As shown in Table 2, compared with the CON and BC birds at 1–21 days of age, supplementation with *L. plantarum* significantly increased the ADFI (*P* < 0.01). Adding *B. coagulans* and *L. plantarum* evidently improved the broilers' ADG as compared to that of the CON broilers at 1–21 days of age (*P* < 0.05). The value of the F/G in the BC treatment was lower than that of the LA treatment (*P* < 0.05).

**Table 2:** Growth performance.

**Item**	**CON**	**BC**	**LA**	**Pooled-SE**	***P*-value**
**1-21 d**					
ADFI (g)	52.97^b^	55.97^b^	61.43^a^	1.057	<0.01
ADG (g)	32.92^b^	36.14^a^	36.31^a^	0.548	0.008
F/G (g/g)	1.618^a,b^	1.549^b^	1.692^a^	0.207	0.008
**22–42 d**					
ADFI (g)	106.3^b^	121.3^a^	126.7^a^	3.223	0.017
ADG (g)	48.75^b^	66.86^a^	69.09^a^	2.572	<0.01
F/G (g/g)	2.181^a^	1.814^b^	1.833^b^	0.565	0.004
**1–42d**					
ADFI (g)	159.2^b^	177.2^a^	188.1^a^	3.872	0.002
ADG (g)	81.66^b^	103.0^a^	105.4^a^	2.439	<0.01
F/G (g/g)	1.950^a^	1.721^c^	1.785^b^	0.001	<0.01

*CON, broilers were not treated except for the base diet; BC, broilers were supplemented with Bacillus coaglulans; LA, broilers were supplemented with Lactobacillus plantarum; ADFI, the average daily feed intake; ADG, the everage daily gain; F/G, feed to gain ratio. The different alphabet in the same line represent significant difference. N = 6*.

In contrast with that of the CON birds, dietary *B. coagulans* and *L. plantarum* markedly improved the ADFI and ADG of the BC and LA birds at 22–42 days of age (*P* < 0.05). Moreover, the F/G of the BC and LA birds was lower than that of the CON birds at 22–42 days of age (*P* < 0.05).

In contrast with that of the CON birds, the effects of *B. coagulans* and *L. plantarum* on ADFI and ADG at 1–42 days of age were consistent with those of the birds at 22–42 days of age (*P* < 0.05). The F/G of the BC and LA birds decreased significantly compared to that of the CON birds at 1–42 days of age, and the F/G of the BC birds was lower than that of the LA birds (*P* < 0.05).

### Effects of Probiotics on Intestinal Injury in Broilers Induced by LPS

**Figure 2:**
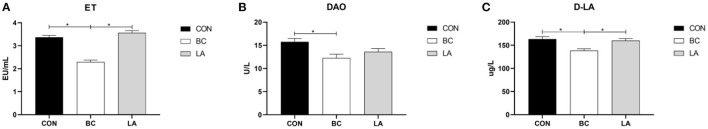
**(A–C)** Effects of probiotics on intestinal injury induced by lipopolysaccharide in broilers. CON, broilers were not treated except for the base diet; BC, broilers were supplemented with *Bacillus coagu-lans*; LA, broilers were supplemented with *Lactobacillus plantarum*; “_*_” means significantly difference. *N* = 6.

To investigate the effect of LPS attack on the intestinal injury of broilers, we detected the ET, DAO, and D-LA contents in the serum. As shown in Figure 2, the levels of ET and D-LA in the BC birds were lower than those in the CON and LA birds (*P* < 0.05, Figures 2A,C), and the level of DAO in the BC birds was lower than that in the CON birds (*P* < 0.05, Figure 2B).

### Effects of Probiotics on Immunoglobulin Content in Broilers Induced by LPS

**Figure 3:**
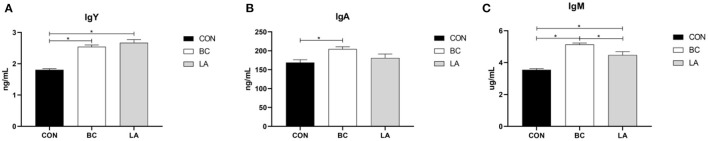
**(A–C)** Effects of probiotics on serum immunoglubins induced by lipopolysaccharide in broilers. CON, broilers were not treated except for the base diet; BC, broilers were supplemented with *Bacillus coagulans*; LA, broilers were supplemented with *Lactobacillus plantarum*; “_*_” means significantly difference. *N* = 6.

As shown in Figure 3, the level of serum IgY in the BC and LA birds was higher than that in the CON birds (*P* < 0.05, Figure 3A). Compared with that of the CON birds, the BC group had an increased serum IgA content (*P* < 0.05, Figure 3B). The level of serum IgM in the BC and LA treatments was higher than that of the CON treatment. Moreover, the IgM content in the CB group was higher than that of the LA group (*P* < 0.05, Figure 3C).

**Figure 4:**
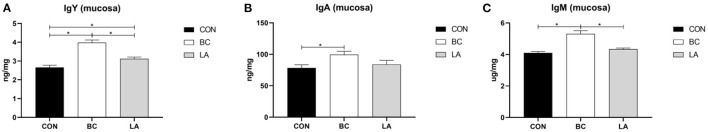
**(A–C)** Effects of probiotics on jejunal mucosa immunoglubins induced by lipopolysaccharide in broilers. CON, broilers were not treated except for the base diet; BC, broilers were supplemented with *Bacillus coagulans*; LA, broilers were supplemented with *Lactobacillus plantarum*; “_*_” means significantly difference. *N* = 6.

In the jejunal mucosa of the broilers, the IgY, IgA, and IgM contents in the CB treatment was significantly higher than that of the CON (*P* < 0.05, Figures 4A–C). In addition, compared with that of the LA broilers, the IgY and IgM levels in the BC group significantly increased (*P* < 0.05, Figures 4A,C). The concentration of IgY in the LA group was significantly higher than that of the CON group (*P* < 0.05, Figure 4A).

### Effects of Probiotics on the Inflammatory Factor Level in the Broilers Induced by LPS

**Figure 5:**
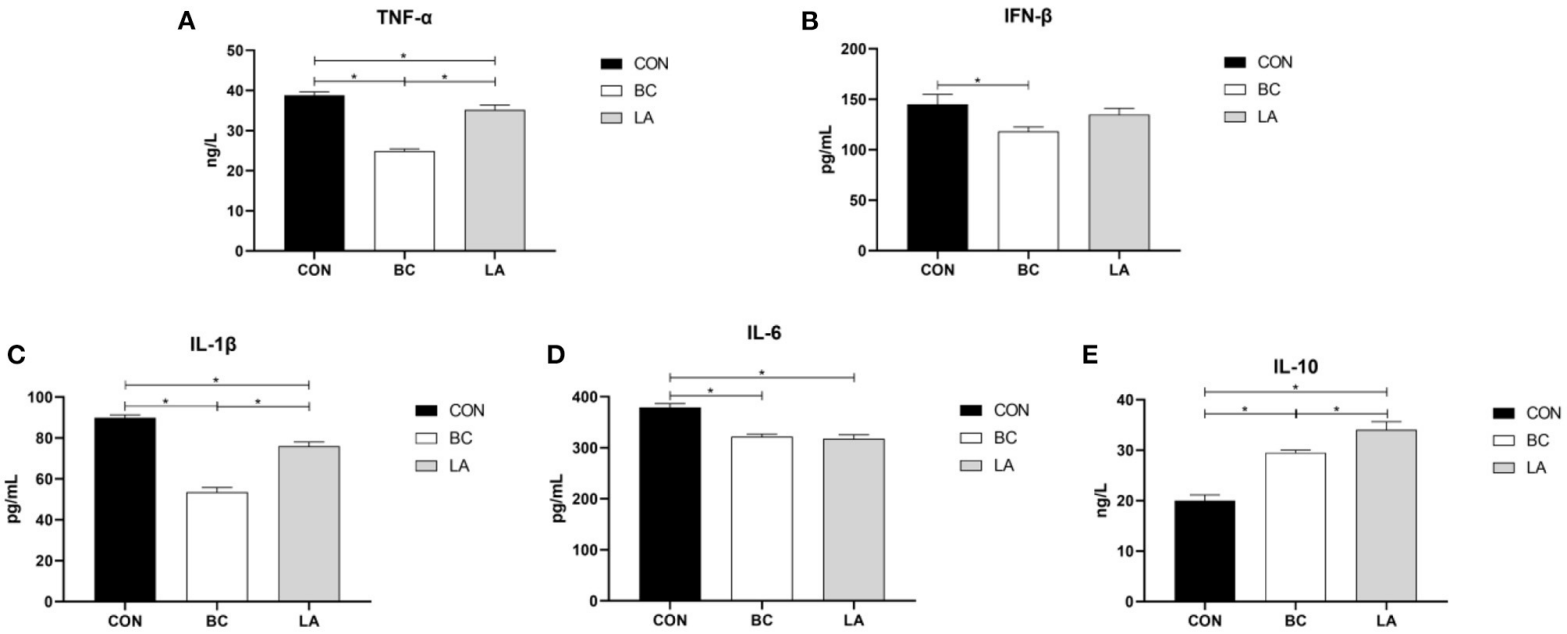
**(A–E)** Effects of probiotics on serum inflammatory factors induced by lipopolysaccharide in broilers. CON, broilers were not treated except for the base diet; BC, broilers were supplemented with *Bacillus coagulans*; LA, broilers were supplemented with *Lactobacillus plantarum*; “_*_” means significantly difference. *N* = 6.

To evaluate the immune effect of *B. coagulans* and *L. plantarum* on broilers challenged by LPS, the inflammatory factors in the serum and jejunal mucosa were individually detected. In contrast with that in the CON treatment, contents of the serum proinflammatory factors (TNF-α, IL-1β, IL-6) in the BC and LA treatments were reduced evidently (*P* < 0.05, Figures 5A,C,D), and the TNF-α and IL-1β contents in the BC treatment were lower than those in the LA treatment (*P* < 0.05, Figures 5A,C). The serum IFN-β content in the BC treatment was lower than that in the LA treatment (*P* < 0.05, Figure 5B). The IL-10 content in the BC and LA treatments was higher than that in the CON treatment (*P* < 0.05, Figure 5E). Moreover, in contrast with that of the BC group, the concentration of IL-10 in the LA group was obviously increased (*P* < 0.05, Figure 5E).

**Figure 6:**
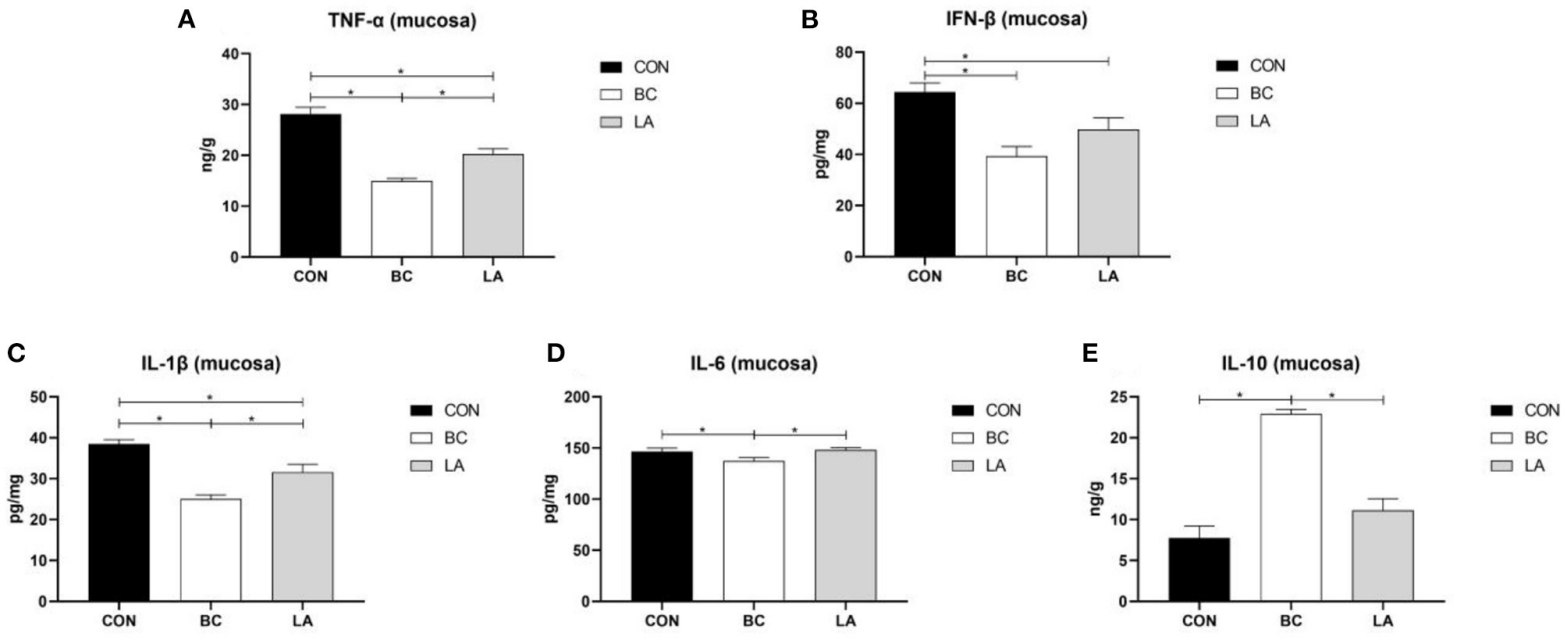
**(A–E)** Effects of probiotics on jejunal mucosa inflammatory factors induced by lipopolysaccharide in broilers. CON, broilers were not treated except for the base diet; BC, broilers were supplemented with *Bacillus coagulans*; LA, broilers were supplemented with *Lactobacillus plantarum*; “_*_” means significantly difference. *N* = 6.

In the jejunal mucosa, TNF-α and IL-1β levels of the CON group were higher than those of the BC and LA groups (*P* < 0.05, Figures 6A,C). Compared with that of the CON birds, the level of IFN-β was decreased significantly in the BC and LA birds (*P* < 0.05, Figure 6B). The level of IL-6 in the BC group was lower than that in the CON and LA groups, whereas the level of IL-1β was higher than that in the other two groups (*P* < 0.05, Figures 6D,E).

### Effects of Probiotics on the Antioxidant Enzyme Activity in the Broilers Induced by LPS

**Figure 7:**
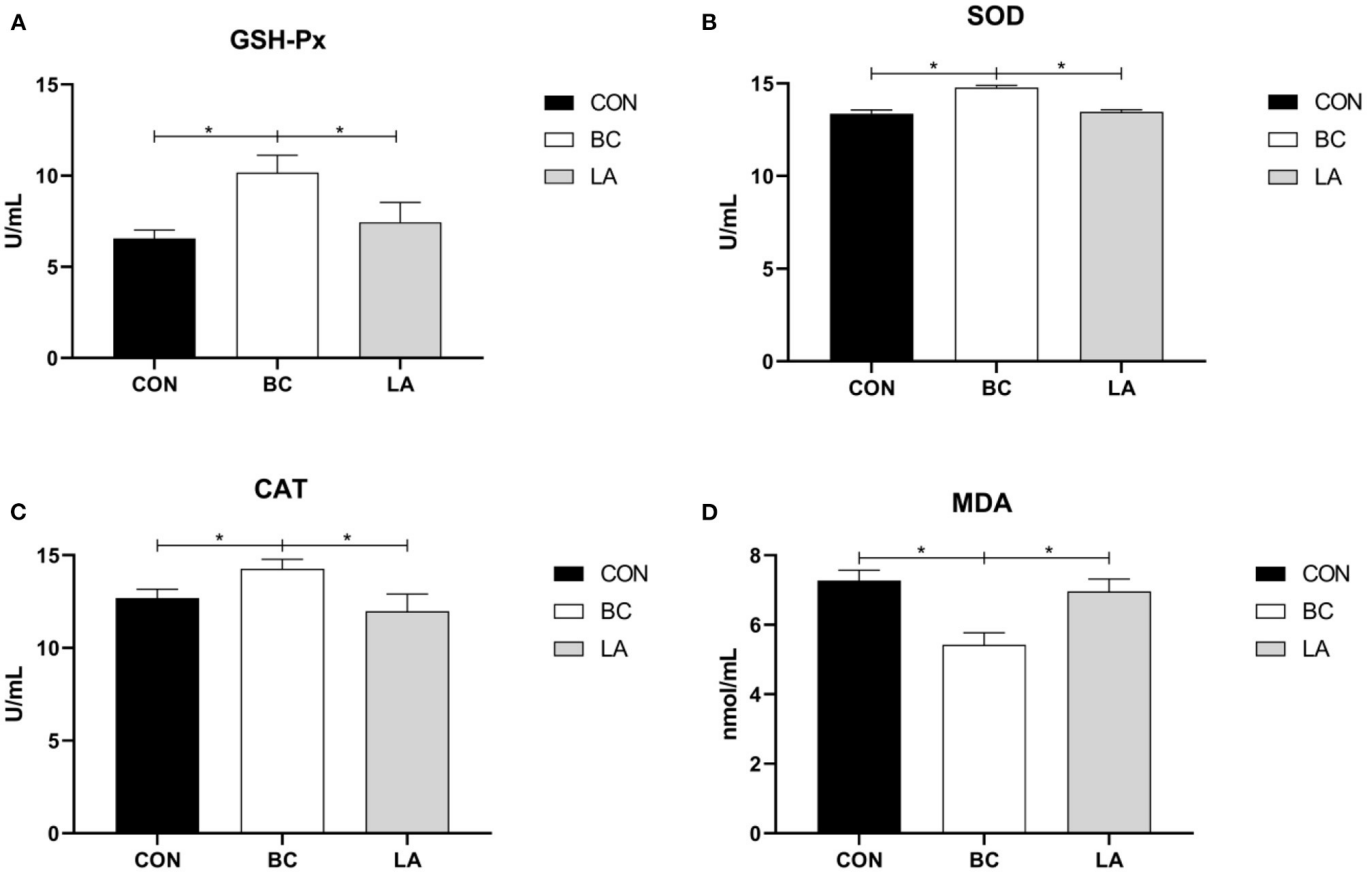
**(A–D)** Effects of probiotics on serum antioxidant enzyme activity induced by lipopolysaccharide in broilers. CON, broilers were not treated except for the base diet; BC, broilers were supplemented with *Bacillus coagulans*; LA, broilers were supplemented with *Lactobacillus plantarum*; “_*_” means significantly difference. *N* = 6.

In Figure 7, the antioxidant enzyme (GSH-Px, SOD, CAT) activities of the BC group were higher than those of the CON and LA groups in the broilers' serum (*P* < 0.05, Figures 7A–C), whereas the level of MDA in the BC group was lower than that in the CON and LA groups (*P* < 0.05, Figure 7D).

### Effects of Probiotics on Gut Microbiota in the Broilers Induced by LPS

**Figure 8:**
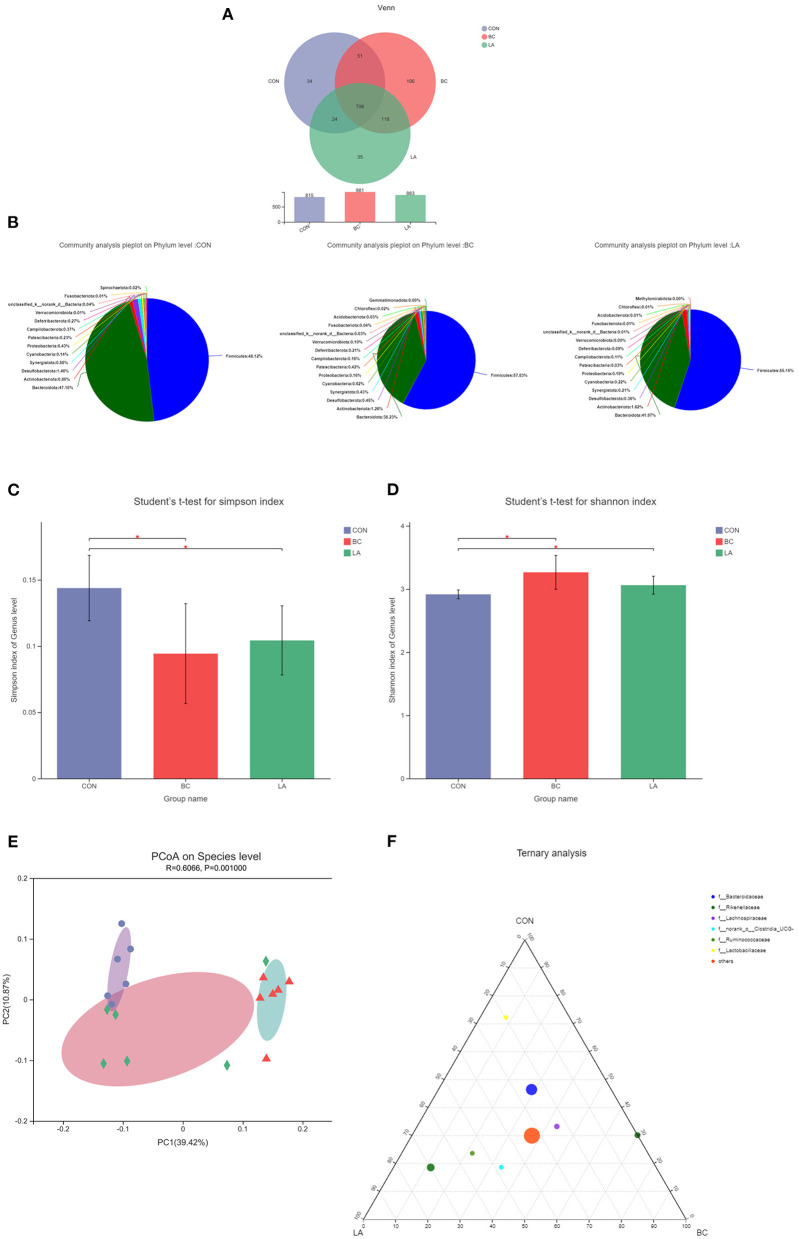
Effects of probiotics on cecal microbiota composition of LPS-attacked broilers. **(A)** OUT, **(B)** community composition, **(C)** simpson index, **(D)** shonnon index, **(E)** PCoA analysis, **(F)** ternary analysis. CON, broilers were not treated except for the base diet; BC, broilers were supplemented with *Bacillus coagulans*; LA, broilers were supplemented with *Lactobacillus plantarum*; “_*_” means significantly difference. *N* = 6.

After the LPS challenge treatment, the OTUs in the BC and LA treatments were higher than that in the CON treatment (Figure 8A). Firmicutes and Bacteroidota were the dominant flora (phylum level), but the proportion of Firmicutes in the CON treatment (48.12%) was lower compared with that in the BC (57.83%) and LA treatments (55.15%) (Figure 8B). The Simpson and Shannon indices indicated that the richness and evenness of the bacterial community of the CON group were significantly different from those of the BC and LA groups (Figures 8C,D, *P* < 0.05). The distance of the PCoA analysis indicated that the species composition of the BC group was different from that of the CON and LA groups (Figure 8E). *Rikenellaceae* were more distributed in the LA and BC groups, and *Ruminococcaceae* were more enriched in the LA group. There was no difference in the distribution of the other levels of microorganisms among the three groups in the ternary phase diagram (Figure 8F).

**Figure 9:**
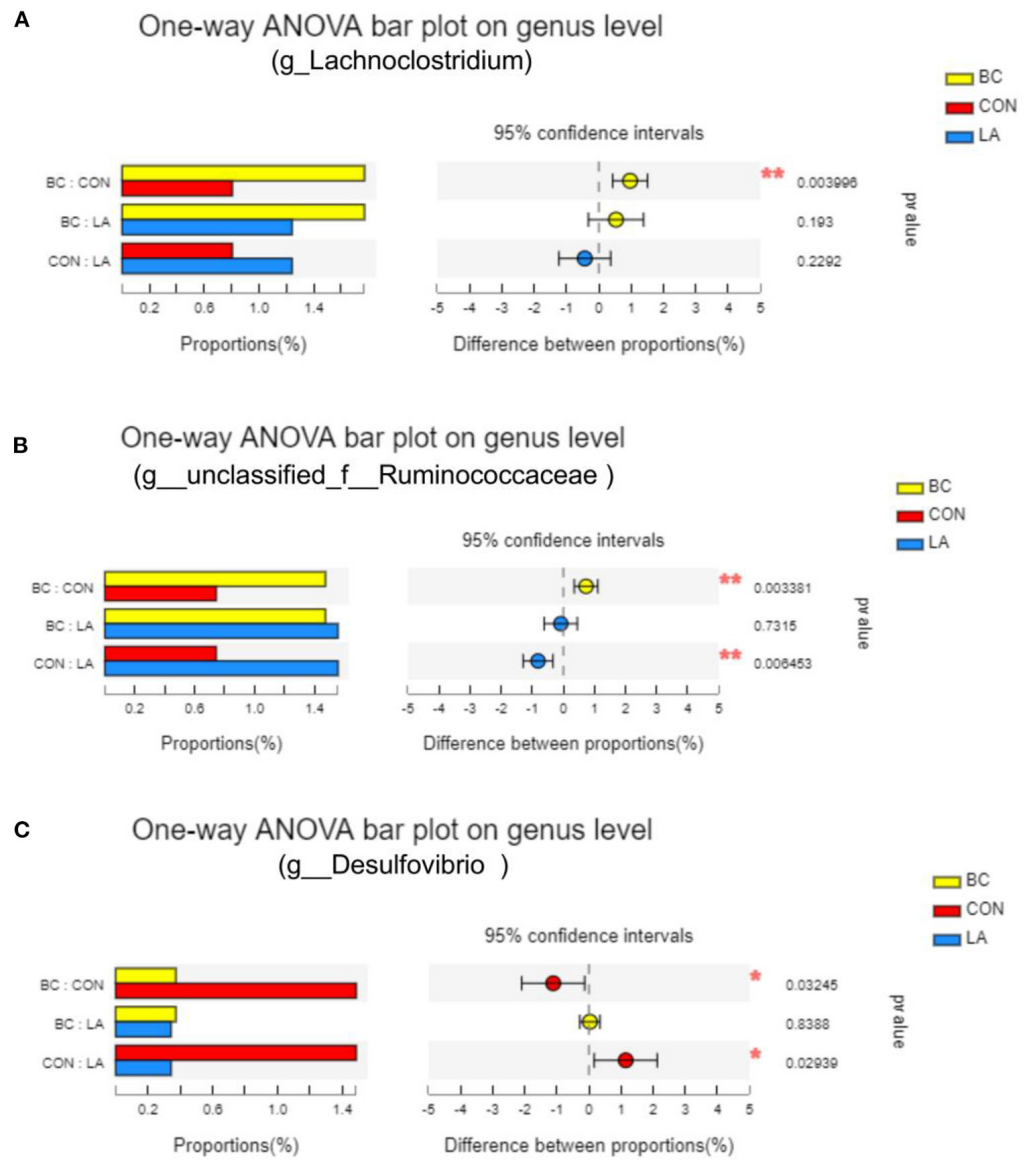
Effects of probiotics on cecal species abundance of LPS-attacked broilers. **(A–C)** Were the test results of the significance of intergroup differences of *Lachnoclostridium, Ruminococcaceae* and *Desulfovibrio*, respectively, inspection methods: one-way ANOVA, correct ways: False discovery rate; CON, broilers were not treated except for the base diet; BC, broilers were supplemented with *Bacillus coagulans*; LA, broilers were supplemented with *Lactobacillus plantarum*; “*” means significantly difference. “_*_” means extremely distinct difference. *N* = 6.

In order to identify the species diversity, we analyzed the species abundance diversity at the genus level, and the intergroup differences were tested for some species (Figures 9A–C). The *Lachnoclostridium* abundance in the BC birds was evidently higher than that in the CON birds (*P* < 0.05, Figure 9A). Compared with the BC and LA treatments, the *Ruminococcaceae* abundance was significantly decreased in the Con treatment (*P* < 0.01, Figure 9B). Moreover, the abundance of *Desulfovibrio* in the CON treatment was significantly reduced compared with that in the BC and LA treatments (*P* < 0.05, Figure 9C).

## Discussion

Many studies have shown that probiotics used in animal husbandry can significantly improve the growth performance of livestock and poultry; for example, adding *L. plantarum B1* to the diet improved the weight gain and feed conversion ratio of broilers (17), while feeding *B. licheniformis* could significantly increase body weight and ADG (18). In our study, feeding *B. coagulans* and *L. plantarum* increased the ADG of the early broilers. Moreover, the addition of two probiotics evidently improved ADG and F/G in later- and full-term broilers. Repeated LPS stimulation reduced the body weight of the birds, while the supplementation of diets with *B. amyloliquefaciens* alleviated the LPS-induced reduction in the ADG of the broilers (19, 20).

Although the weight of the broilers after LPS stimulation were not recorded due to experimental errors, it could be inferred from the results of this study and those of previous studies that the addition of *B. coagulans* and *L. plantarum* could improve the growth performance of broilers (17, 18), which may benefit the resistance of broilers to anti-inflammatory consumption.

The D-LA mainly originates from bacterial production in the intestinal tract and is commonly used as a marker of bacterial infection. A higher concentration of intestinal bacteria implies that more D-LA is produced. The DAO activity is associated with the maturation and integrity of the small intestinal mucosa, and DAO activity is a useful biomarker for estimating the severity of intestinal mucosal disorders (21). It has been reported that D-LA level, ET level, DAO activity were increased in intestinal mucosa damage (22). Our study showed that *B. coagulans* evidently reduced the levels of ET, DAO, and D-LA in the serum of broilers challenged by LPS. This is similar to the effect of other probiotics, that is, the triple live agents of *Bifidobacterium, Lactobacillus*, and *Streptococcus thermophilus* decreased the DAO activity, and D-LA and ET contents in rats undergoing cardiopulmonary bypass (22). Moreover, supplementation with *L. salivarius* significantly reduced the serum D-LA and DAO contents of LPS-attacked piglets (23). However, *L. plantarum* had no obvious effect in our experiment. The data suggested that dietary *B. coagulans* could reduce LPS-induced intestinal mucosal injury.

The beneficial effects of probiotics *in vivo* have been proven, for example, increased peripheral immunoglobin production stimulated IgA secretion (24). As the biggest producer of immunity *in vivo*, the intestinal tract produces a large amount of IgA by its activated mucosal B cells, which plays the role of the first-line immune defense (25). In this study, the contents of IgA, IgY, IgM were increased in the broilers' serum and jejunal mucosa after *B. coagulans* supplementation. These indicated that the immunity of broiler chickens fed with *B. coagulans* was enhanced by regulating the caecal microbiota, wherein *B. coagulans* helped to resist the infection stress. Supplementation of *L. plantarum* increased the levels of IgY and IgM in the serum and jejunal mucosa, suggesting that dietary *L. plantarum* had a positive effect on the immunity of broilers, but the expression of different immunoglobulins may vary. The contents of IgY and IgM (including serum and jejunal mucosa) in the BC birds were higher than that in the LA birds, which indicated that *B. coagulans* was more beneficial than *L. plantarum* in improving the immunity of broilers in our study.

When animals received repeated LPS stimulation, the peripheral blood immune organs such as spleen and thymus significantly proliferated, produced inflammation and pro-inflammatory cytokines (TNF-α) (26, 27). Mazkour et al. (28) demonstrated that the combination of *B. coagulans* and *B. subtilis* significantly reduced the level of the serum inflammatory cytokine TNF-α, which was induced by Salmonella. In addition, *B. coagulans* downregulated the expression of the pro-inflammatory cytokine IFN-γ, and it is believed that *B. coagulans* had potential to suppress intestinal inflammation in broilers challenged by *S. enteritidis* (29). In the present study, dietary *B. coagulans* significantly decreased the contents of the pro-inflammatory factors TNF-α, IL-1β, IL-6, and IFN-β in the serum and jejunum mucosa of the broilers. Supplementation with *L. plantarum* reduced the levels of the pro-inflammatory factors TNF-α and IL-1β in the serum and jejunal mucosa. Dietary supplementation of the two probiotics increased the contents of anti-inflammatory factors (IL-10) in the serum. Moreover, *B. coagulans* increased the IL-10 content of the broilers (in serum and jejunum mucosa) and was superior to *L. plantarum* in terms of immunogenicity. The results showed that adding probiotics into the broilers' diet could activate the immune system of the broiler, which could help them resist LPS stimulation.

Lipopolysaccharide stimulation not only easily led to intestinal inflammation, but also often caused acute liver injury (30, 31). Probiotics are an important factor affecting the oxidative status of the gut by exhibiting direct antioxidant properties and inducing the intrinsic organisms signaling antioxidant defense (32). Chorawala et al. (33) showed that probiotics could resist LPS-induced oxidative stress by reducing the MDA content and restoring the glutathione content in the colon. Besides, our previous study proved that *B. coagulans* elevated the serum CAT, SOD, and GSH-Px activity levels and decreased the serum MDA content in conventionally grown broilers (34). *B. coagulans* increased the levels of GSH-Px, SOD, and CAT, decreased the levels of MDA, and it performed better than *L. plantarum* in this study. These results were consistent with previous studies, indicating that *B. coagulans* could reduce LPS-induced oxidative stress injury, while the antioxidation of *L. plantarum* was not ideal.

At the phylum level, Bacteroidetes and Firmicutes were the dominant bacterial groups, which was consistent with previous studies in which Bacteroidetes and Firmicutes constituted most of the microbial communities in chickens at the phylum level, and these bacteria are known to play a role in energy production and metabolism (35–37). The ternary phase diagram helped to prove this point. The Simpson and Shannon indices of the probiotics treatment indicated that the colonization of probiotics had no adverse effect on the intestinal microflora of broilers and increased the community richness of intestinal flora.

In this study, the enrichment of the genus *Lachnoclostridium* was significantly increased by the addition of *B. coagulans*. Many species belonging to the genus are producers of butyrate (38), which is associated with the gut barrier, inflammation, and endotoxin levels (39). Combing with the above indices of mucosal injury, it could be concluded that *B. coagulans* attenuated inflammation, and resisted the intestinal barrier injury by increasing the *Lachnoclostridium* abundance in the LPS-challenged broilers' intestinal tract. The content of ET, DAO, D-LA in the LA group were not significantly different from those in the CON group, correspondingly. There was no significant difference in the *Lachnoclostridium* richness between the two groups (CON and LA). On the other hand, supplementation with *L. plantarum* improved some of immune and antioxidant properties of the broilers attacked by LPS. It's suggested that the way through which *L. plantarum* activated host immunity in this trial may not be through regulating the *Lachnoclostridium* abundance.

*Bacillus. coagulans* increased the abundance of *Ruminococcaceae* in LPS-challenged broilers. Interestingly, Ma et al. (40) reported that the increased abundance of *Ruminococcaceae* due to *B. subtilis* addition was associated with increased ADG and body weight. This could explain the significant improvement in the ADG and F/G of the BC and LA groups. Moreover, *Desulfovibrio* is an inflammatory-promoting taxon of bacteria associated with anxiety and depression (41). One recent study showed that probiotics (*L. rhamnosus LS-8* and *L. crustorum MN047*) manipulated the gut microbiota by decreasing the abundance of *Desulfovibrio* and increasing *Lactobacillus* and *Bifidobacterium*, thereby reducing the circulating LPS levels (42). In this study, both *B. coagulans* and *L. plantarum* supplementation evidently reduced the abundance of *Desulfovibrio*.

## Conclusion

In conclusion, diets supplemented with *B. coagulans* and *L. plantarum* improved the growth performance of broilers under LPS stimulation and alleviated the mucosal injury, inflammatory response, and oxidative stress, which may be related to changes in the intestinal flora caused by the addition of probiotics. Findings from our study demonstrate the potential applications of *B. coagulans* and *L. plantarum* in poultry, specifically its beneficial effects in the performance of chickens, which is of great significance because of the increasing demand for poultry meat. The specific mechanism needs further in-depth study.

## Data Availability Statement

The original contributions presented in the study are included in the article/supplementary material, further inquiries can be directed to the corresponding author.

## Ethics Statement

The study was conducted according to the guidelines of the Animal Management Rules of the Ministry of Health of the People's Republic of China, and approved by the Ethics Committee of Zhejiang Agricultural and Forestry University, Hangzhou, China.

## Author Contributions

YY: conceptualization and writing—original draft preparation. QL: methodology and data curation. XZ: software and visualization. YX: validation and formal analysis. KJ: investigation. JL: resources and funding acquisition. GC: project administration. YY and GC: writing—review and editing. YX and KJ: supervision. All authors have read and agreed to the published version of the manuscript.

## Funding

This study was supported by Zhejiang Leading Innovation and Entrepreneurship Team (No. 2020R01015); the National Natural Science Foundation of China (No. 32002195); Zhejiang Key Agricultural Enterprise Research Institute (No. 2021Y30004); and the Nature Science Foundation of Zhejiang Province (No. LQ20C170003).

## Conflict of Interest

XZ and JL was employed by Zhejiang Vegamax Biotechnology Co., Ltd. The remaining authors declare that the research was conducted in the absence of any commercial or financial relationships that could be construed as a potential conflict of interest.

## Publisher's Note

All claims expressed in this article are solely those of the authors and do not necessarily represent those of their affiliated organizations, or those of the publisher, the editors and the reviewers. Any product that may be evaluated in this article, or claim that may be made by its manufacturer, is not guaranteed or endorsed by the publisher.
